# Correction: The mechanism of β-N-methylamino-L-alanine inhibition of tRNA aminoacylation and its impact on misincorporation

**DOI:** 10.1016/j.jbc.2022.102544

**Published:** 2022-10-04

**Authors:** Nien-Ching Han, Tammy J. Bullwinkle, Kaeli F. Loeb, Kym F. Faull, Kyle Mohler, Jesse Rinehart, Michael Ibba

The article included typographical errors due to overseeing font/symbol conversation. Concentrations of enzymes used in deacylation assay were 1 **micro**M and the authors provide the correct legend for figure 4 as follows.

“**Figure 4. BMAA perturbs AlaRS editing.** A deacylation assay using [^3^H]Ser-tRNA^Ala^ is carried out with 1 **μM** HsAlaRS, 1 **μM** HsAlaRS with 50 mM BMAA, 1 **μM** C723A, and a no enzyme control. The percentage of misacylated tRNA remaining in the reaction is calculated at each time point. The counts of time point zero was set as 100% for the diacylation analysis. All of the experiments were done at least three times, and the error bars indicate the standard deviations of the means.”

The correct concentration of AlaRS enzyme in deacylation assay described in the **Materials and methods** “*Misaminoacylation and deacylation*” section was also 1 **micro**M and a correct sentence on page 1408 should read as follows.

“Deacylation reactions were carried out at 37 °C in aminoacylation buffer containing [^3^H]Ser-tRNA^Ala^ and 1 **μM** AlaRS.”

The authors apologize for the mistakes.

